# A greedy regression algorithm with coarse weights offers novel advantages

**DOI:** 10.1038/s41598-022-09415-2

**Published:** 2022-03-31

**Authors:** Clark D. Jeffries, John R. Ford, Jeffrey L. Tilson, Diana O. Perkins, Darius M. Bost, Dayne L. Filer, Kirk C. Wilhelmsen

**Affiliations:** 1 Renaissance Computing Institute, University of North Carolina, Chapel Hill, NC USA; 2 Perspectrix, Pittsboro, NC USA; 3 Psychiatry, University of North Carolina School of Medicine, Chapel Hill, NC USA; 4 Genetics, University of North Carolina School of Medicine, Chapel Hill, NC USA; 5 Neurology, University of North Carolina School of Medicine, Chapel Hill, NC USA; 6 Neurology, West Virginia University Rockefeller Neuroscience Institute, Morgantown, WV USA

**Keywords:** Computational models, Diagnostic markers, Predictive markers, Prognostic markers, Computational neuroscience, Computational biology and bioinformatics, Neuroscience, Biomarkers

## Abstract

Regularized regression analysis is a mature analytic approach to identify weighted sums of variables predicting outcomes. We present a novel Coarse Approximation Linear Function (CALF) to frugally select important predictors and build simple but powerful predictive models. CALF is a linear regression strategy applied to normalized data that uses nonzero weights + 1 or − 1. Qualitative (linearly invariant) metrics to be optimized can be (for binary response) Welch (Student) t-test p-value or area under curve (AUC) of receiver operating characteristic, or (for real response) Pearson correlation. Predictor weighting is critically important when developing risk prediction models. While counterintuitive, it is a fact that qualitative metrics can favor CALF with ± 1 weights over algorithms producing real number weights. Moreover, while regression methods may be expected to change most or all weight values upon even small changes in input data (e.g., discarding a single subject of hundreds) CALF weights generally do not so change. Similarly, some regression methods applied to collinear or nearly collinear variables yield unpredictable magnitude or the direction (in p-space) of the weights as a vector. In contrast, with CALF if some predictors are linearly dependent or nearly so, CALF simply chooses at most one (the most informative, if any) and ignores the others, thus avoiding the inclusion of two or more collinear variables in the model.

## Introduction

Regularized regression modeling strategies for choosing variables and weights are mature and extensively documented^1^. Ordinary (linear) least-squares (OLS) minimizes the mean of squared differences (MSE) between the N real entries of a targeted response vector versus a weighted linear combination of p predictors, that is, the matrix product Xβ where X is a N-by-p matrix and β is the weight (column) vector. Seeking optimal weights for OLS dates to the early 1800s and works of Legendre and Gauss. However, OLS can provide uninterpretable and unstable weights when exact or approximate collinearity exists between predictors or when problems are underdetermined (i.e., N << p). Regularized regression may employ Lagrange multipliers to constrain the optimization; examples include Tikhonov regularization (ridge regression, using constrained L2-norm), least absolute shrinkage and selection operator (basic LASSO, using constrained L1-norm), and elastic net (using constrained convex sum of the L1- and L2-norms)^1–4^. Each method has its advantages and disadvantages. As one advantage, LASSO regression with L1-norm includes choice of a parameter called s that can indirectly force a solution to have few nonzero weights, enabling cross-referencing of selected predictors and a search for underlying meaning among them^5^. Thus, discovering small sets of collectively informative predictors may suggest causal networks. However, regarding instability due to predictors that are collinear or nearly so, various classifiers including LASSO regression are susceptible and must be modified accordingly^6^. But overall, the widely acknowledged value and general use of LASSO algorithms^7–11^ recommends comparisons with LASSO as the “gold standard”.

We present “Coarse Approximation Linear Function” (CALF), an algorithm that, applied to some examples of real data, builds models with frugal use of predictors, superior qualitative metric values versus LASSO, as well as superior permutation test performance. CALF inherently precludes collinearity issues and provides better consistency among selected predictors when applied ~ 1000 times to, say, random 90% subsets of subjects, a test called herein “popularities”. These properties could yield relatively simple causal interpretation of the interplay of predictors. Furthermore, the improvements in some cases could mean the difference between finding statistical significance or noting a mere trend.

CALF has already been applied in three publications that aimed to discover variables measured at initial clinical presentation that collectively increased prediction of transition to psychosis within two years in a cohort of persons at elevated risk^12–14^. In those papers CALF was sketched and heavily used, but few underlying details were provided. An improved program now yields updates of those early findings.

## Methods

CALF assumes predictor values are corrected for possible confounders and then converted to z-scores. Binary response vectors (e.g., control versus case) are represented as 0 or 1 values; otherwise, continuous response vectors are converted to z-scores. CALF uses a greedy forward selection algorithm to accumulate a set of nonzero weights, each ± 1. As the calculation proceeds, an N-dimensional response vector Y is compared to the product of N-by-p data matrix X and tentative p-dimensional weight vector β with − 1, 0, or + 1 components; comparison uses a chosen metric (p-value, AUC, or correlation). In the initial step all β entries are 0, and one by one each is changed to ± 1 (− 1 may be needed initially to make correlation values positive). The metric values of all 2p possible choices are noted and the first (in order of matrix columns) such choice that is at least as good as any subsequent choice is kept. In subsequent iterations the remaining 0 entries in β are changed one by one to ± 1, and the first such choice, if any, among 2(p − 1) possibilities that improves the metric and does so at least as well as any subsequent choice is kept; and so on. If at any iteration there is no such choice to improve the metric, then CALF ends. Else, if the number of nonzero β entries reaches a preset limit L, then CALF ends. Else, if no unselected predictors remain, then CALF ends. This completely different from LASSO and is also different from simply choosing L predictors with the best metric values; in fact, CALF does not necessarily choose those. Of course, as a greedy algorithm, CALF could become “stuck” on a local optimum and ignore a global optimum (Supplement 1).

Subject to its limits, CALF seeks optimized metric values meaning: a Welch t-test p-value decreasing from default = 1; an AUC increasing from default = 0.5; or a correlation increasing from default = 0. Note that only the direction in p-space of the vector β matters for these three metrics; β could be rescaled by any positive multiplier without affecting them. The fact that only direction of β matters partly explains why CALF works with simple ± 1 weights.

Thus, basic CALF is a simple greedy algorithm. When certain predictors are highly correlated CALF chooses the one, if any, from unselected predictors with the greatest improvement in model performance. For example, suppose a data matrix and metric (metric = Welch-Student p-value, AUC, or Pearson correlation) leads to a CALF solution. Suppose we form a new data matrix by copying the matrix to double the number of predictors (columns) causing perfect collinearity. Applying CALF will result in the same solution. CALF simply chooses the first (or neither) of the two copies of any predictor. Suppose instead of a copy of the data matrix we adjoin a new matrix of the same size but with values all slightly perturbed from the true values with refreshed z-scores (near collinearity). CALF typically chooses the same number of predictors, a mix of the original and the modified, but at most one of each pair. That is, with sufficiently small perturbations, at most one of the paired predictors will be used. Since nonzero weights are all ± 1, wild values of weights are not possible. This is illustrated in Supplement 2.

Pseudocode for CALF follows.

Data:

N-by-1 response vector Y (column matrix).

N-by-p data matrix X (one column for each predictor).

Parameters:

Natural number L ≤ p, a limit on the number of predictors to be employed in a solution.

Result:

p-by-1 weight vector β (column matrix) with at most L nonzero entries (weights) =  ± 1.

Let d^k^ denote the kth column of diag(1).
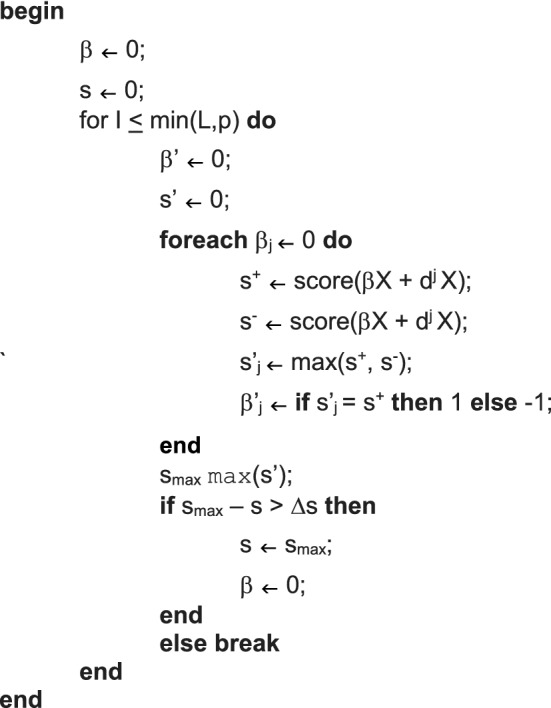


### Model selection and comparison

When evaluating models, we seek simplicity, goodness of fit, permutation test performance, and consistency of predictor popularities. Our goals are:

1. Simplicity: Select a relatively small set (e.g., at most 20) collectively informative predictors from a larger set of potential predictors.
2. Goodness of fit: Seek good Welch p-value, AUC, or correlation, as appropriate or preferred.
3. Permutation performance: Compute an empirical p-value from permutation tests and reject algorithms scoring above 0.05 as ineffective.
4. Popularity: Reapply the algorithm to many (e.g., 1000) 90% random subsets of subjects and seek a pattern with a “cliff”, that is, a small number of selected predictors that occur frequently while other predictors occur seldom or not at all. If the response is binary, each selection uses ~ 90% of each group (control and case).

The second goal assumes appreciation of the strengths and sometimes subtle shortcomings of the metrics. However, just achieving this goal does not preclude overfitting.

The third goal—and also the first—may be served by permutation tests. Permutations and calculations of metric values yield an empirical p-value (not to be confused with the metric Welch p-value). This is a type of probability value well-known in permutation testing^15^. We recall that empirical p-value testing entails applying a given algorithm to many (e.g., 1000) pseudo-data versions of a classification or approximation problem in which all components of the response vector have been randomly permuted. The empirical p-value is estimated from the empirical number E of times the algorithm performance is by chance superior in D applications to permuted data vs. application to the true data^16,17^; specifically, empirical p-value = (E + 1)/(D + 1). Thus, the empirical p-value of a given algorithm is an estimate of an upper limit of the risk that an apparently good metric value has been achieved by chance.

The fourth goal is consistent marker popularity. That is, we may select many (e.g., 1000) random subsets of true data (e.g., 90%) and apply CALF to each with a limiting number of nonzero weights such as 20. A histogram of selection counts may then reveal frequently selected predictors (e.g., > 300 times in 1000 trials). Avoiding infrequently selected predictors may help avoid overfitting and use of predictors of small effect size. Ideally, the popularities of selected predictors should display a cliff, that is, a sharp decrease in popularities for predictors not in a small pool. Thus, a cliff might suggest an optimal L value, enabling the first goal.

Sweeping through the CALF limit L = 1, 2,…, 20 generally reveals a particular choice of L with minimal empirical p-value. If two choices have about the same empirical p-value, then the one with smaller L is preferred. Popularity histograms can also contribute to the selection of L. Similarly, sweeping through a range of s values in LASSO logistic regression or basic LASSO may suggest indirectly an optimal number of employed predictors.

Given a limit L in CALF, the particular CALF solution with L as limit is called CALFL; likewise, LASSOs is defined. CALFL and LASSOs mean versions of the algorithms with L in the range 1, 2,…, 20 and s values in some range that indirectly yields roughly one to 20 nonzero LASSO weights. For binary response vectors, we compare CALF vs. LASSO logistic regression with AUCs over the ranges.

### Five examples of data sources and their analyses

We illustrate the performances of CALF and LASSO with five data sets drawn from three previously reported studies and two publicly available sources (access for all five is in our Implementation). We utilized the R package Lasso and Elastic-Net Regularized Generalized Linear Models (glmnet)^4^ to run LASSO. All figures were prepared with Excel® and PowerPoint® in Microsoft 365®.

The first four example have diverse predictor types and various proportions of N and p, but all have binomial response vectors. LASSO logistic regression (family = “binomial”) is appropriate and widely used for such data^7–11^. It uses a function of the regression sum itself, the response, inverse of log of 1 plus the exponential of the same predictor sum, and a weight penalty term. The algorithm seeks to minimize the function^3^ (see a helpful introduction by Hastie, Qian, and Tay at https://glmnet.stanford.edu/articles/glmnet.html). For our fifth example with its continuous response vector (age of onset), we compare basic LASSO (family = “gaussian”) versus CALF in terms of Pearson correlation. We also “adjust” CALF with a simple intercept value and common multiplier of ± 1 weights to enable comparison with LASSO using mean squared error (MSE). Our present goal is realistic mathematical illustration only—not generation of medical hypotheses requiring extensive knowledge of the diseases mentioned. Example parameters are in Table 1.

**Table 1:** Parameters of five data examples.

Example	N		p Predictors	Response vector	Predictor	CALF metric	LASSO metric
	Controls	Cases					
1	40	32	135	Binary	Blood analytes	p-value	Composite function
2	38	30	130	Binary	Leukocytic miRNAs	AUC	Composite function
3	40	32	19	Binary	Symptom severity	p-value	Composite function
4	213	165	140	Binary	Blood analytes	p-value	Composite function
5	NA	582	9312	Continuous	SNP allele coding	Correlation	Composite function

As shown in the figures, AUC, empirical p-value, and predictor popularities were consistently used to directly compare CALF vs. LASSO performance.

## Results

Again, CALF assumes predictor values are corrected for possible confounders and then converted to z-scores. While binary response vectors are represented as 0 or 1, continuous response vectors are converted to z-scores. In basic CALF, metric performance means goodness of Welch t-test p-value (p-value), AUC, or Pearson correlation (correlation). We show in this section how use of seemingly primitive ± 1 weights can outperform LASSO logistic regression or basic LASSO.

### Example 1

We utilized a dataset from the North American Psychosis-Risk Longitudinal Study^18^. Here the goal is use of blood plasma analyte levels to predict future development of psychosis in subjects meeting research criteria for psychosis high-risk. Data included levels of 135 blood analytes from 72 subjects including 40 who did not and 32 who did subsequently convert to frank psychosis. The 135 blood analyte levels were determined from samples taken at the time of study enrollment. The CALF solution here differs from that of the original publication^12^ (different metric).

**Figure 1:**
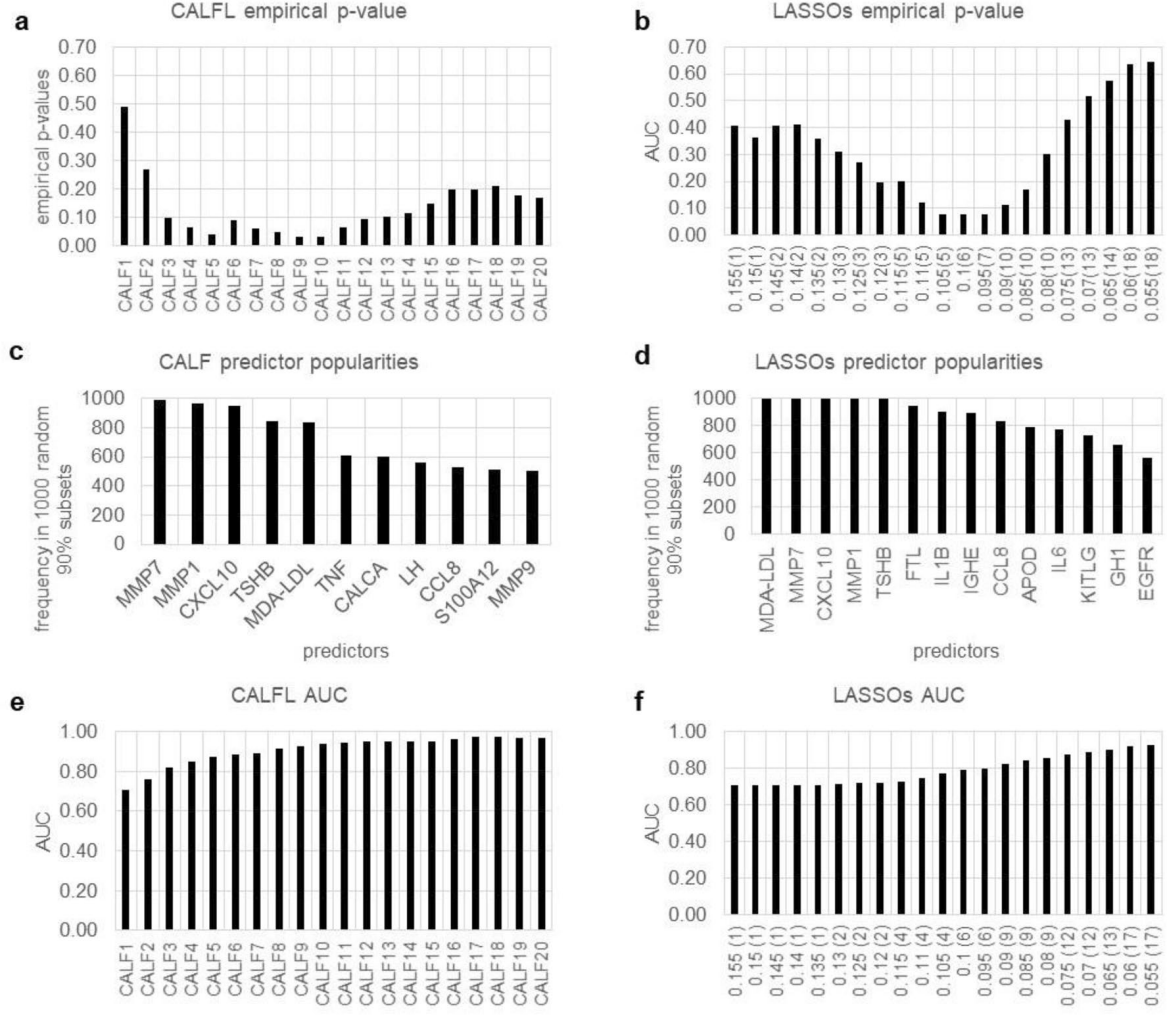
Comparison of CALF and LASSO logistic regression results for Example 1. (**a**) For CALF, empirical p-value is 0.040 with five predictors, that is, CALF5. (**b**) For LASSO, there are no s values for which LASSOs selects 4, 8, 9, 11, 12 14, 15, 16, 17, or 20 predictors. Otherwise, the lowest empirical p-value 0,076 occurs with s = 0.10 (six predictors). (**c**) A popularity cliff for CALF occurs at five predictors but (**d**) there is no comparable cliff for LASSO. (**e**) CALF5 AUC is 0.873, but (**f**) LASSOs does not match this until s reaches 0.075 with 12 predictors.

The CALF metric for this example was p-value of each CALF sum vs. group membership. A general comparison of CALF and LASSO for this example appears in Fig. 1. Since CALF5 and LASSO.075 have about the same AUC (~ 0.873), we show them explicitly.

$$ {\text{CALF5 }} = {\text{ MMP7 }} + {\text{MDA}} - {\text{LDL }} - {\text{MMP1 }} + {\text{TSHB }} - {\text{CXCL1}}0 $$

$$ {\text{LASSO}}.0{75 } = \, - 0.{417 } + 0.{494}*{\text{MMP7 }} + 0.{237}*{\text{MDA}} - {\text{LDL }} - 0.{228}*{\text{MMP1 }} + 0.{184}*{\text{TSHB }} - 0.{181}*{\text{CXCL1}}0 \, + 0.{159}*{\text{FTL }} + 0.0{76}*{\text{CCL8 }} + 0.0{54}*{\text{IGHE }} + 0.0{37}*{\text{APOD }} + 0.0{24}*{\text{KITLG }} + 0.0{11}*{\text{TTR }} - 0.00{1}*{\text{IL6 }} + 0.00{1}*{\text{IL1B}} $$

(The analyte symbols are defined in the earlier publication^12^. CALF predictors are in order of choice, and LASSO predictors are in order of decreasing weight magnitude. In LASSO.075 only three of ten decimal places from glmnet R are shown for brevity).

Note the predictors in CALF5 appear with the same signs and the same order in the first five of LASSO.075; however, LASSO.075 requires seven additional predictors to reach the AUC of CALF5.

*Comparison* Simplicity, empirical p-values, consistency of predictor choices, and AUCs all suggest CALF is a superior algorithm for this example.

Lastly, Supplement 3 documents examination of the same example by several conventional algorithms. Using mostly default but some selected parameters did not in any of those methods appear to offer reliable classification.

### Example 2

The second example employs another dataset from the North American Psychosis-Risk Longitudinal Study^18^. Here the goal is use of leukocytic microRNA (miRNA) levels from a blood draw upon initial presentation of clinical high-risk subjects to predict a subsequent conversion to frank psychosis. Assayed were levels of 130 leukocytic miRNAs from 68 subjects, 38 who did not and 30 who did convert. This time our CALF solution used AUC as metric. (Welch p-value was used in the original publication^19^).

**Figure 2:**
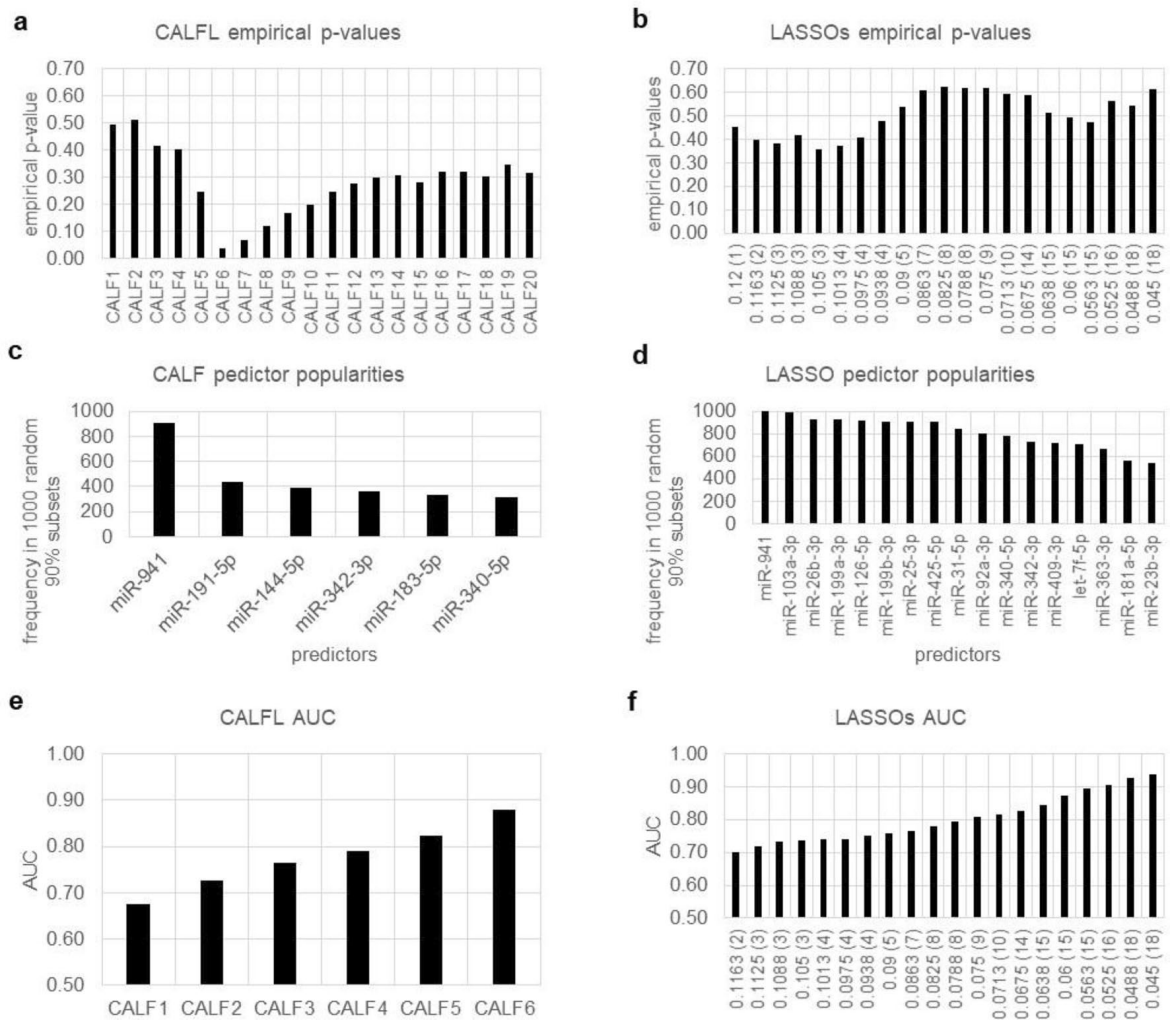
Comparison of CALF and LASSO logistic regression results for Example 2. (**a**) CALF6 attains an empirical p-value of 0.040 while (**b**) the lowest for LASSO over the shown range is 0.359. (**c**) CALF will not select more than six predictors, so the popularity graph is not informative. (**d**) LASSO fails to show a cliff in popularities. (**e**) For AUC, CALF6 attains 0.878 while (f) all LASSO AUCs require at least 15 nonzero weights to achieve the same.

With the AUC metric and 100% of true data CALF chooses at most six predictors (CALF6 AUC = 0.878) (Fig. 2e). The AUC for LASSO continues to increase as more variables are added (up to the limit of 20 predictors in Fig. 2f), even as the empirical p-value decreases, indicating overfitting.

The CALF solution with empirical p-value 0.040 (Fig. 2a) is

$$ {\text{CALF6 }} = \, + {\text{miR}} - {941 } - {\text{miR}} - {25} - {\text{3p }} - {\text{miR}} - {\text{378a}} - {\text{3p }} + {\text{miR}} - {34}0 - {\text{5p }} + {\text{miR}} - {221} - {\text{3p }} - {\text{miR}} - {\text{26b}} - {\text{3p}} $$

There is no LASSO solution with an empirical p-value < 0.05 (Fig. 2b). In contrast with CALF, predictor popularities for LASSO showed less variability and no obvious “cliff” (Fig. 2c,d).

*Comparison* Empirical p-values favor the CALF solution.

### Example 3

We utilized a third dataset from the North American Psychosis-Risk Longitudinal Study^18^. This time predictors are discrete but nonbinary, and there are more subjects than available predictors (N > p). Data included severity ratings of 19 symptoms from the Scale of Prodromal Symptoms (SOPS)^4,15^ determined at initial presentation from 72 high-risk subjects, 40 who did not and 32 who did convert later to a psychosis diagnosis. The process in this example differed from the original publication^13^ in that we utilized the same subjects as in example 1 rather than the entire cohort.

**Figure 3:**
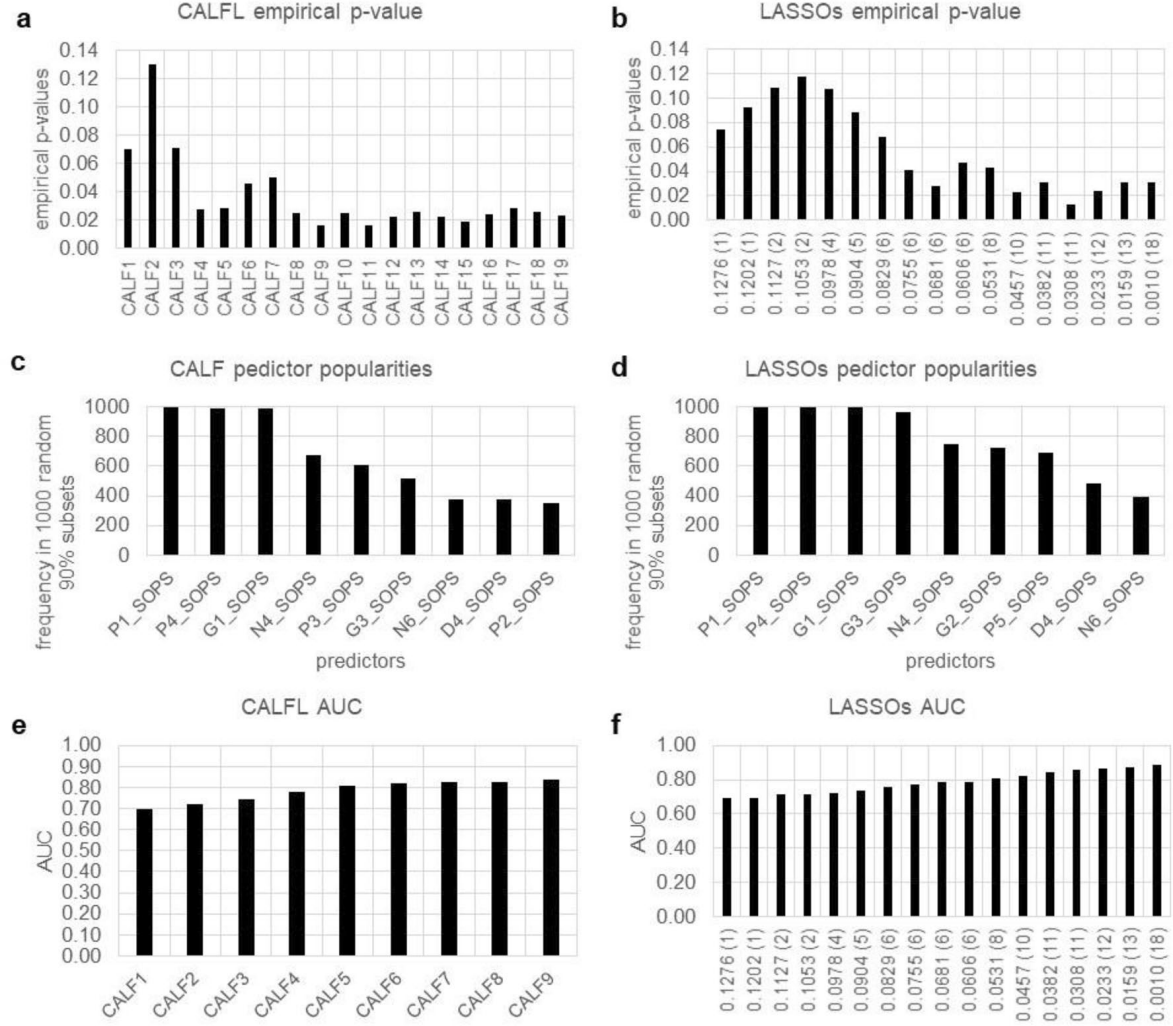
Comparison of CALF and LASSO logistic regression results for Example 3. (**a**) CALF4 empirical p-value is 0.027 while CALF9 yields 0.019. Simplicity favors CALF4. (**b**) Noting the different vertical scale, the lowest empirical p-value 0.065 for LASSO occurs with s = 0.15 and only one predictor—a mere trend. (**c**) CALF has a strong cliff at three predictors, but empirical p-values of recommend CALF4. (**d**) LASSO has a strong cliff with four predictors but an unacceptable empirical p-values for two or more predictors. (**e**) CALF4 has AUC 0.777 while (**f**) LASSO only exceeds that at LASSO.068 by using six predictors and overfitting.

As shown in Fig. 3a and b, symptom P1 (unusual thought content) was the most informative. However, using only P1 yields a poor empirical p-value (about 0,070). This illustrates the fact that one-by-one metric values in might not be the best approach. CALF4 and CALF9 had much better empirical p-values (0.027, 0.016, respectively) and yielded AUCs of 0.777 and 0.839, respectively (Fig. 3e). The first LASSO solution with an empirical p-value significantly < 0.05 is LASSO.0681 with p-value of 0.028; it uses six predictors and has an AUC of 0.784 (Fig. 3f). Predictor popularities for CALF and LASSO showed similar variability and both evidenced a “cliff” (Fig. 3c,d). The two solutions are:

$$ {\text{CALF4 }} = + {\text{P1 }} + {\text{G3 }} - {\text{P4 }} + {\text{G1}} $$

$$ {\text{LASSO}}.0{68 } = \, - 0.{24}0 \, + \, 0.{396}*{\text{P1 }} - 0.{21}0*{\text{P3 }} + 0.{186}*{\text{G1 }} + 0.{119}*{\text{G3 }} + 0.0{44}*{\text{P5 }} + 0.0{3}0*{\text{G2}} $$

*Comparison* Simplicity and AUC values favor the CALF solution.

### Example 4

This example employs a dataset from Alzheimer disease (AD) research reported in the Biomarkers Consortium Project publication “Use of Targeted Multiplex Proteomic Strategies to Identify Plasma-Based Biomarkers in Alzheimer’s Disease” (https://fnih.org/our-programs/biomarkers-consortium/programs/plasma-based-biomarkers-alzheimers). The goal is use of blood plasma analyte levels to predict risk of future development of dementia in persons meeting research criteria for mild cognitive impairment. Data included levels of 140 blood analytes from 378 subjects, 213 who were not and 165 who were subsequently diagnosed with dementia. The 140 blood analyte levels were determined from samples taken at the time of study enrollment.

**Figure 4:**
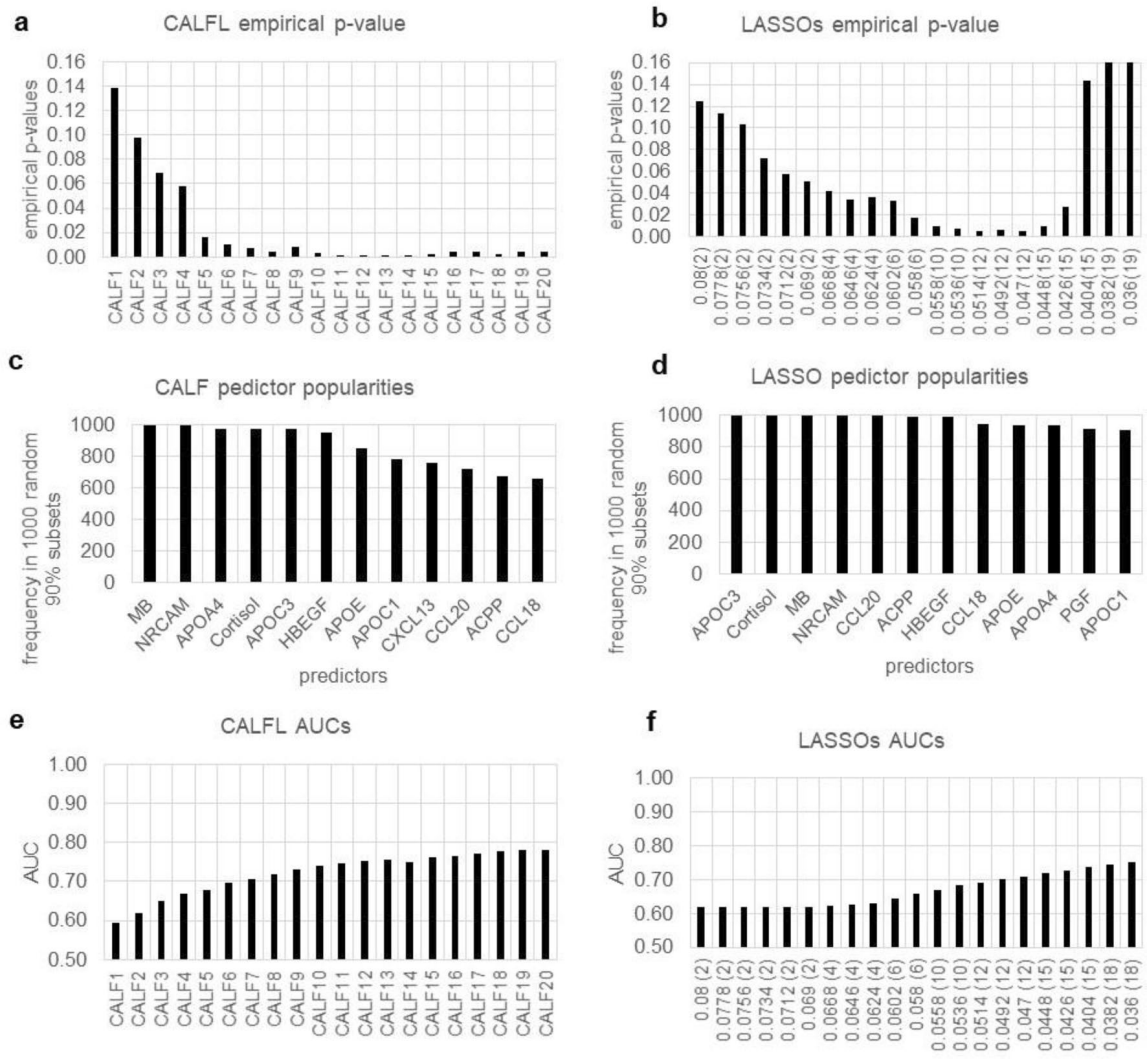
Comparison of CALF and LASSO logistic regression results for Example 4. (**a**) CALF6 has an empirical p-value 0.010. (**b**) Comparable empirical p-values for LASSO require at least 10 predictors, risking overfitting. (**c**) A cliff occurs at six predictors for CALF, but (**d**) LASSO has no comparable cliff. (**e**) For CALF6 the AUC is a weak 0.696, but (**f**) LASSO requires at least 10 predictors to achieve the same, risking overfitting.

Figure 4 provides the empirical p-values, predictor popularities, and AUC values for the CALFL and LASSOs models. Interestingly, among the eight most popular with CALF are four apolipoproteins (APOC3, APOA4, APOE, APOC1); LASSO lists only one in its top eight.

Both CALF and LASSO solutions achieved empirical p-values < 0.05. The CALF6 empirical p-value = 0.010 (Fig. 4a) and AUC = 0.696 (Fig. 4e) were like the LASSO.0558 using ten predictors with p-value = 0.010 (Fig. 4b) and AUC = 0.670 (Fig. 4f), with the solutions:

$$ {\text{CALF6 }} = {\text{ APOC3 }} - {\text{CCL2}}0 \, + {\text{Cortisol }} - {\text{NRCAM }} + {\text{MB }} - {\text{HBEGF}} $$

$$ {\text{LASSO}}.0{558 } = - 0.{257 } - 0.{1}0{2}*{\text{APOC }} + {3 }0.0{8}0*{\text{CCL2}}0 \, + 0.0{36}*{\text{NRCAM }} + 0.0{33}*{\text{ACPP }} - 0.0{27}*{\text{Cortisol }} - 0.0{17}*{\text{MB }} + 0.00{3}*{\text{CCL18 }} - 0.00{1}*{\text{APOC1 }} + 0.00{1}*{\text{PGF }} + 0.000*{\text{CRP}} $$

Note that the CALF popularities (Fig. 4c) exhibit at cliff but LASSO popularities (Fig. 4d) do not.

*Comparison* Simplicity favors selection of the CALF solution.

### Example 5

Data are from the National Institute on Aging and Center from inherited Disease Research of Johns Hopkins University at https://www.ncbi.nlm.nih.gov/projects/gap/cgi-bin/study.cgi?study_id=phs000168.v2.p2. Data include age of onset (ranging from 52 to 98 years) from 582 AD patients. Predictors are minor allele/major allele coding reflected in 9312 SNPs, scored as − 1 (homozygous minor allele), 0 (heterozygous), 1 (homozygous major allele); each SNP then converted to a z-score. Age of onset itself is also recoded as a z-score.

The primary CALF goal is selection of a ± 1 weighted sum of a small set of SNPs that has a high Pearson correlation with age of onset. A byproduct is an “adjusted CALF” designated adjCALF obtained by applying OLS to the simple case that the CALF solution itself is the one predictor, and the response is unchanged. The adjCALF enables MSE value calculation vs. LASSO. In more detail, OLS yields adjCALF = b + m(Xβ) where m (a common, positive multiplier) and b (a single intercept value repeated in a column vector) are derived as scalars from OLS applied to Y and Xβ = CALF solution. That is, b is a N-by-1 matrix with all entries b, and m > 0 is a real multiplier of all entries in the N-vector Xβ.

**Figure 5:**
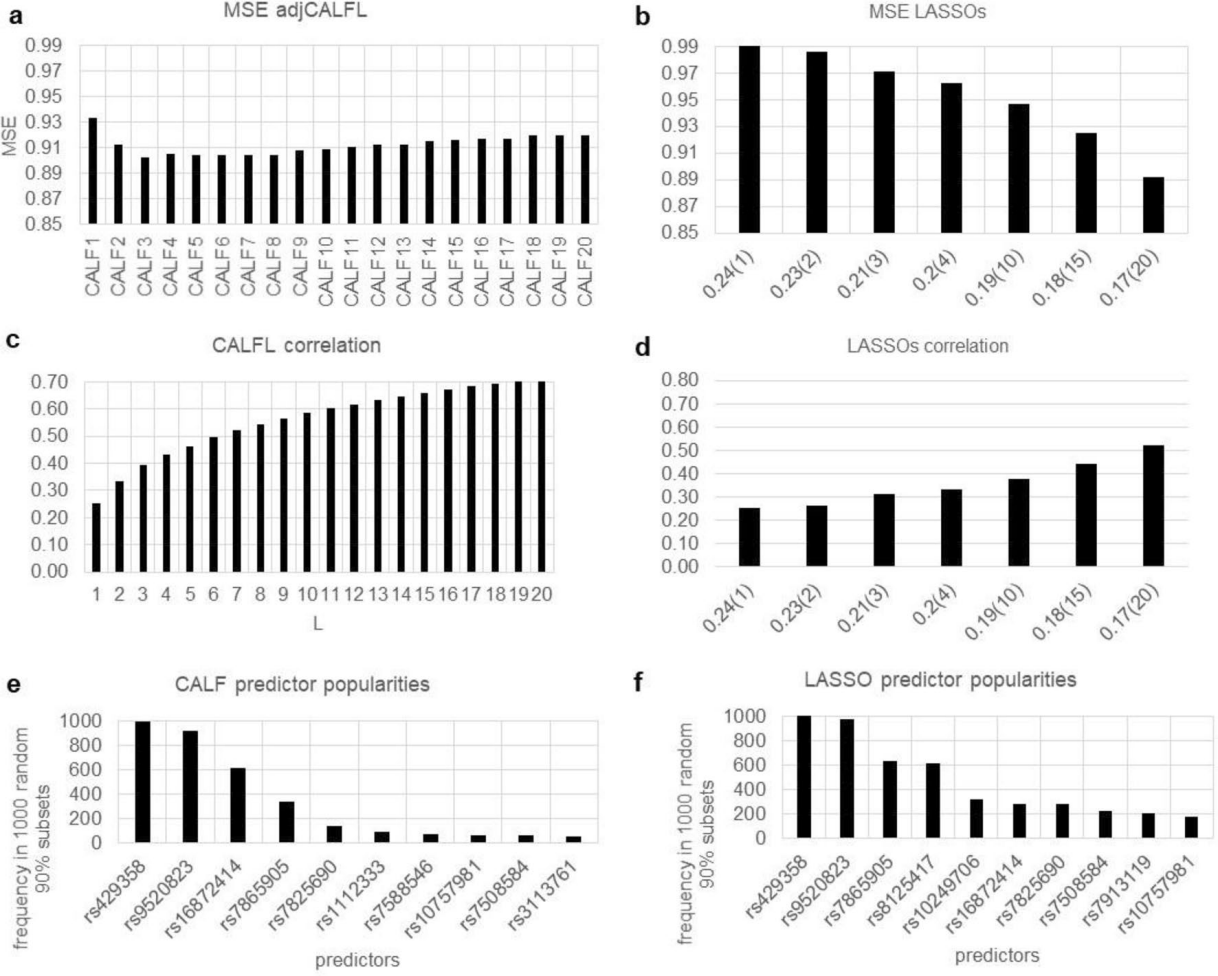
CALF versus LASSO for example 5. (**a**) MSE of adjCALFL reaches minimum 0.902 with three weights or 0.905 with four. (**b**) Only seven distinct numbers of nonzero weights are possible over the s scan with MSE falling from 0.991 to 0.892, including 0.962 with four weights, (**c**) The correlations for CALFL over L from 1 to 20 increase from 0.254 to 0.717 with 0.433 for CALF4. (**d**) For LASSO correlations increase from 0.254 (same as CALF1) to 0.524, including 0.332 for LASSO.2 (at four weights). (**e**) CALF popularities (same as adjCALF) for 1000 random 90% subsets of true data show a drop from more than 300 to under 150 beyond four predictors. (**f**) LASSO popularities also show a cliff beyond a slightly different choice of top four predictors, but the popularities of additional predictors are much larger, indicating ambiguity of choices.

Regarding empirical p-value calculations using the correlation metric for CALF, we have observed a perfect value (1/1001) for CALFL over 1000 random permutations with L = 1 to 20 predictors. In fact, the true correlation of CLAF4 was observed to be ten or more standard deviations above averages of correlations using permuted data. Likewise, LASSO solutions have perfect empirical p-value scores. Consequently, the results presented in Fig. 5 do not include those results. Instead, MSEs, correlations, and popularity profiles are shown.

Also, LASSO scans over s yield only solutions with seven numbers of predictors: 1, 2, 3, 4, 10, 15, or 20. Going from two to three predictors and three to four yields improvements in MSE of − 0.0082 and − 0.0088 per predictor (Fig. 5b). However, going next from four (s = 0.2) to 10 (s = 0.19) predictors yields a smaller − 0.0025 improvement per predictor. As shown in detail in Fig. 5, the simplicity and performance of CALF4 recommend it over LASSO.2.

Individually, the correlations of the first two SNPs are − 0.25 and + 0.23, the highest in magnitudes of all 9312 SNPs. As expected, the leading SNP rs429358 (https://www.ncbi.nlm.nih.gov/snp/?term=rs429358); this is one of two SNPs that define the APOE ε4 variant, a well-known genetic risk factor for AD. The protein APOE is a lipid carrier in the central and peripheral nervous systems^20^. The APOE ε4 variant is said to have “unparalleled influence on increased late-onset AD risk”^21^ (late-onset predominates in AD). The SNP rs429358 has alleles T>C and is in exon 4 of gene APOE https://www.ncbi.nlm.nih.gov/snp/rs429358. In our normalization system (0/0 → − 1, 0/1 → 0, 1/1 → + 1, thence to z-scores), the C/C allele maps to a negative z-score. Thus, the CALF4 weight − 1 is consistent with increasing CALF4 function values with age and hence, stronger correlation with age of onset and association with risk of late-onset AD, as expected.

Representative CALF4, adjCALF4, and the LASSO.2 solutions (using four predictors) are:

$$ {\text{CALF4 }} = \, - {\text{rs429358 }} + {\text{rs952}}0{823 } + {\text{rs16872414 }} - {\text{rs78659}}0{5} $$

$$ {\text{adjCALF4 }} = \, 0 + 0.0{6292}*{\text{CALF4}}. $$

$$ {\text{LASSO}}.{2 } = \, - {3}.{\text{61E}} - 0{6 } - {5}.{\text{23E}} - 0{2}*{\text{rs429358 }} + {2}.{\text{67E}} - 0{2}*{\text{rs952}}0{823 } - {1}.{\text{67E}} - 0{3}*{\text{rs78659}}0{5} - {9}.{\text{91E}} - 0{4}*{\text{rs8125417}} $$

The four LASSO weights are ordered by decreasing magnitude. Note that first three LASSO.2 predictors are also among the four predictors in CALF4 in the same order and with the same signs. LASSO.2 simply chooses the four predictors with strongest individual correlation magnitude. However, only six predictors are in both the 20 predictors of CALF20 and the 20 predictors of LASSO.17, underscoring the differences in the algorithms’ predictor choices.

**Figure 6:**
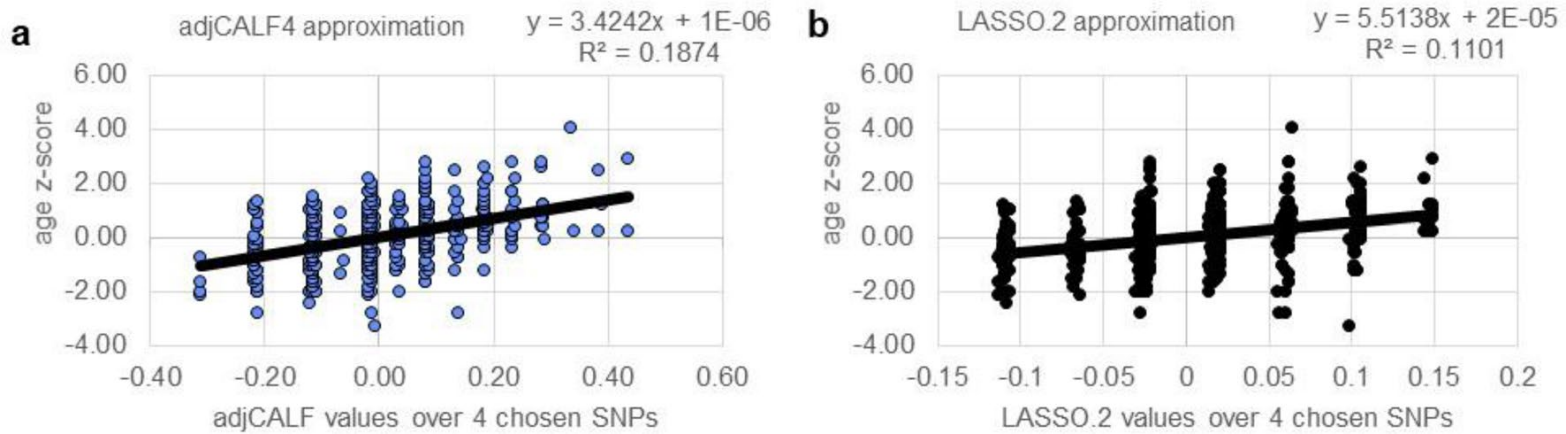
A visual comparison of CALF4 versus LASSO.2 approximations of age of onset as a z-score. (**a**) The correlation of CALF4 is reflected in trendline R^2^ = 0.187. (**b**) For LASSO.2, R^2^ = 0.110.

The constraint of using four predictors leads to a visual comparison of the algorithms. Each SNP attains three values in the data matrix, so four SNPs could have up to 81 combinations; different choices lead to different numbers of combinations with different numbers of distinct algorithm values. We observed sets of 60 distinct CALF4 values and 72 LASSO values; the sets cluster differently, as shown in Fig. 6.

The second-most popular SNP in both CALF4 and LASSO.2 solutions was rs9520823; the 1/1 variant was associated with later age of onset (yielding a positive regression weight). This SNP is in an intron of gene ABHD13, a ubiquitously expressed gene in the ABHD family with protein products important in lipid synthesis and degradation^22,23^. If the rs429358 SNP is deleted from the data matrix, rs9520823 becomes first chosen and CALF4 correlation decreases from 0.433 to 0.421 (data not shown). That is, after discarding APOE, this ABHD13 SNP leads CALF4 choices and correlates almost as well with age of onset. Investigation of causality involving APOE and ABHD13 functions in AD is suggested by these findings. Lipid transport, reception, and metabolism are active AD research arenas^24^.

**Figure 7:**
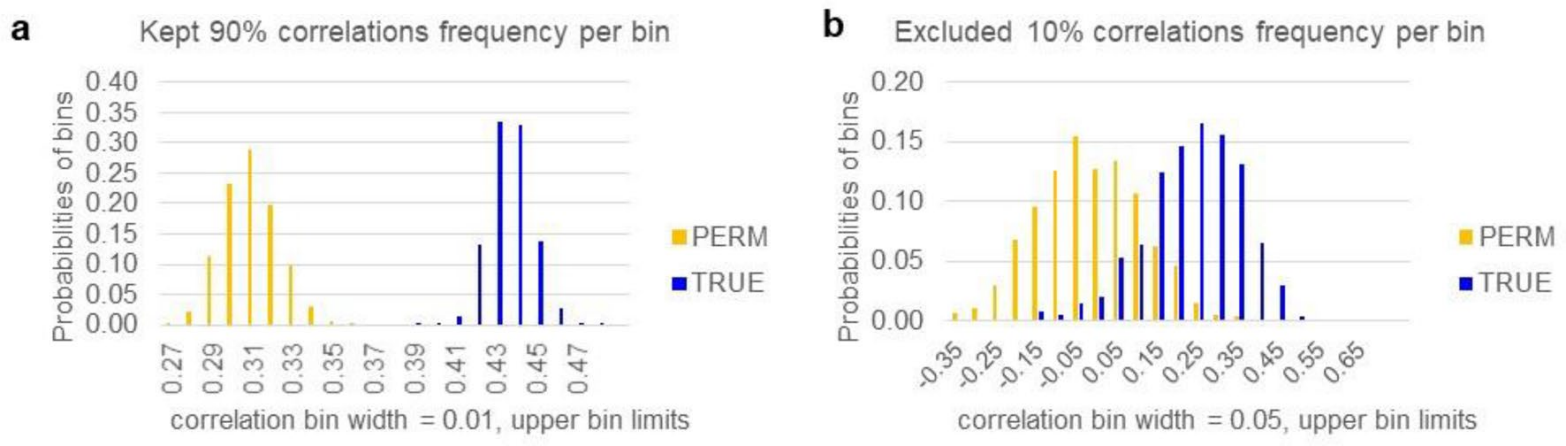
Cross-validation analysis. (**a**) A histogram for cross-validation CALF4 on random 90% kept subsets of SNP data in 1000 trials includes bins of correlation values versus ages of onset. TRUE bars denote values of correlations using 1000 random (with replacement) 90% subsets. PERM bars denote first a random permutation of all 582 ages of onset, then a repetition of the TRUE analysis. Thus, PERM is the null hypothesis result, that is, the absence of information. Welch t-test p-value of the two sets of correlations is ~ 0.00. (**b**) Shown is a histogram of bins of correlation values with ages of onset. TRUE bars denote values of correlations using 1000 random 10% subsets complementary to each of the subsets in (**a**). PERM denotes first a random permutation of all 582 ages of onset, then a repetition of the TRUE calculation. Thus, PERM is the null hypothesis result. Normal distributions of these TRUE and PERM excluded values have K–S goodness of fits α = 0.05 and 0.20 (so the PERM distribution is very close to normal). The average of PERM correlations in the trials was ~ 0.006, but the average of TRUE correlations was ~ 0.26. Assuming normality of both sets, Welch t-test p-value (two-sided) is ~ 5.45E−307.

Regarding cross-validation performance, we applied CALF with a limit of four nonzero weights to 1000 random 90% subsets of true data for training and then applied each of those 1000 solutions to the 1000 complementary 10% subsets, recording the correlation values. The distribution of 1000 cross-validation results is shown in Fig. 7. We also randomly permuted the response vector and then repeated the cross-validation process.

*Comparison* We conclude that CALF4 achieves strong cross-validation in the permutation test sense for this example. Because using the s parameter in LASSO does not generally determine the number of nonzero weights in random subset solutions, analogous analysis of LASSO demanding simplicity (a fixed number of nonzero weights) is not possible.

## Discussion

**Figure 8:**
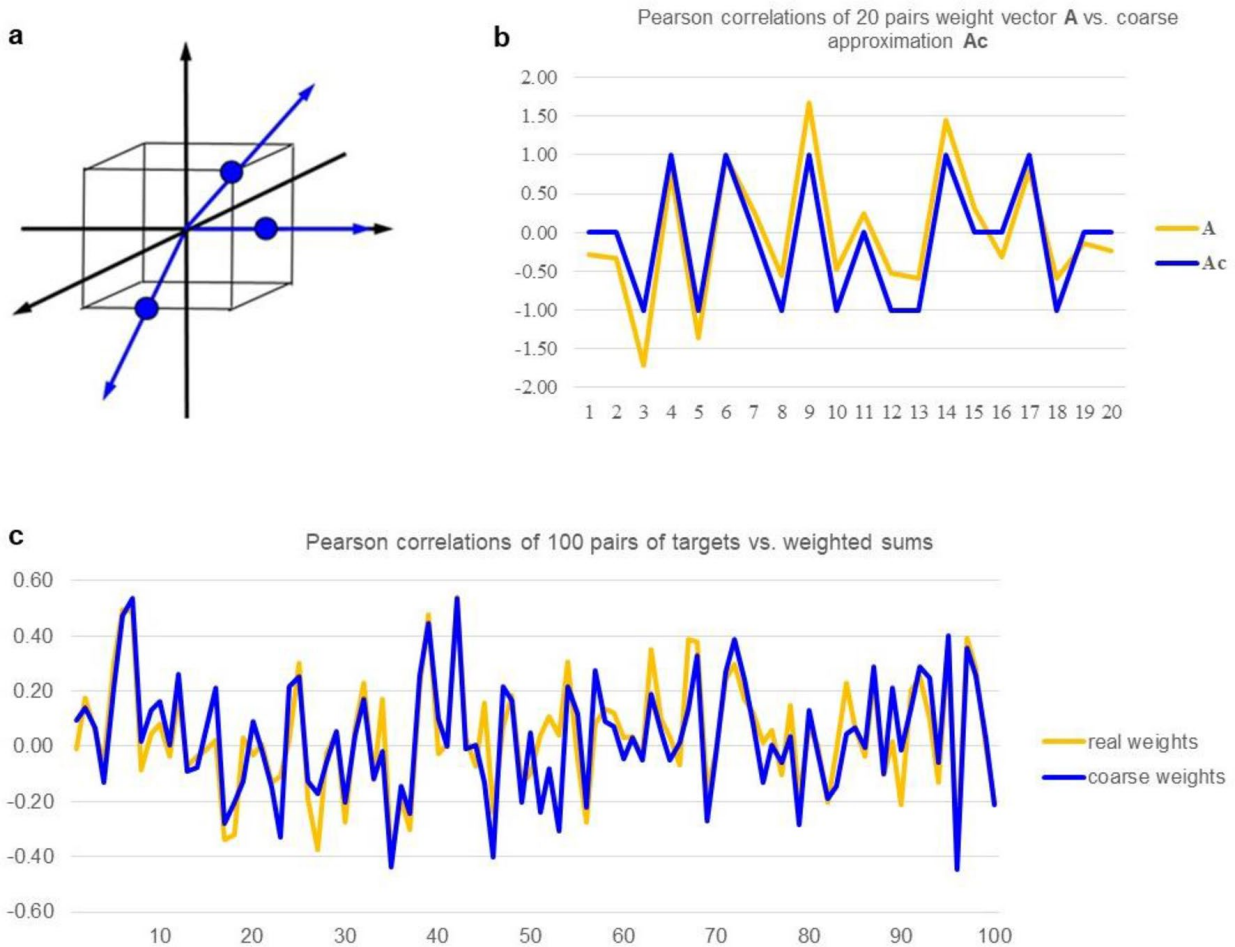
Geometry of CALF and simulations of coarse approximations. (**a**) Three rays (of 26 possible) employed by CALF in 3-dimensional space relate to coordinates of the vertices of the cube (the eight triplets of ± 1). One ray passes through a vertex, one through the midpoint of an edge, and one through the midpoint of a face. (**b**) Visual representation of a typical, single coarse approximations of a real 20-dimensional vector. The real vector **A** has 20 normally distributed components, mean = 0 and sd = 1; components of the coarse approximations **Ac** use threshold =  ± 0.4307. That is, if a component of **A** is < 0.4307, then the corresponding component of **Ac** is − 1, and so on. (**c**) Among 20-dimensional vectors, a plot of 100 comparisons of dot products of **A** with **B** versus **Ac** with **B**, where **A** and **B** are normally distributed, and **Ac** is the coarse approximation of **A**.

Components of the CALF function are geometrically suggested and numerically tested in Fig. 8. The 2-dimensional surface area of the cube in 3-dimensional space in Fig. 8a is 24, so the ratio of the number of possible CALF rays to area is 26/24. Generalizing the concepts to n-dimensional space there are 3^n^-1 rays passing from the origin through all points with coordinates that are combinations ± 1 or 0 (excepting the origin itself). The (n − 1)-dimensional area of the surface of the n-cube is n2^n^. Thus, the ratio of the number of all possible CALF rays to surface area is (3^n^ − 1)/(n2^n^). It can be shown using calculus that in dimensions ≥ 4, this ratio exceeds 1.05^n^. Roughly speaking, the rays available to CALF approximations, each representing a direction available for the approximation, become "exponentially crowded" on the surface of the n-cube as dimension increases.

Figure 8b shows that normally distributed vector components are fairly tracked by coarse approximations, implying the directions of the two vectors in 20-dimensional space are similar. Figure 8c shows that correlations of a normally distributed vector or its coarse approximation with an independent 20-dimensional vector (such as a response vector in a regression analysis) are little different.

In any linear regression model, a metric compares the N-dimensional response vector with the product of the data matrix and a calculated weight vector, possibly with addition of an intercept value. For the Welch p-value, AUC, or correlation scores, only the *direction* of the weight vector is significant. How the weight vector is calculated is the subject of CALF; LASSO and some other algorithms do not directly optimize these metrics. Furthermore, empirical p-values, simplicity, and popularity of chosen predictors in random subsets must be considered along with metric performance. This why CALF might outperform some other algorithms.

Another facet of CALF is combinatorial. For example, from the first data set, five predictors were chosen from 135. There about 3.47E8 choices possible of five among 135; if each of five nonzero weights can be ± 1, then a total of 1.11E10 combinations (directions in 135-space) are available.

Regarding computational cost of CALF, a run of the algorithm with limit L and p predictors may require an order of p*L sums. One illustration of the closeness of a linear function of this term to total cost is in Supplement 4.

Of course, upon finding a CALF solution, the coarse weights could always be adjusted (say, by gradient descent) to try to improve metric value, permutation tests, and subset consistency. But seeking to improve all three would be a slippery slope indeed; the utility of CALF is its simplicity, not ultimate precision with a given data set. Research time might be better spent seeking from completely different directions (e.g., etiological) some companion rationale for the CALF selection of predictors.

In addition to modifications by Tolosi and Lengauer^6^ regarding collinearity, many other authors have contributed novel versions of LASSO. For example, Meinshausen^25^ invented a two-stage procedure termed the relaxed LASSO for sparse, high-dimensional data that may address biasing toward zero of LASSO estimates. A full survey of all published modifications of LASSO is beyond our scope; we merely present a completely different regression algorithm.

## Conclusions

CALF deterministically seeks a coarse sum of a few predictors to optimize a metric. As with any multiple regression approach, the goal of CALF is discovery of a network of informative predictors, not identification one by one of markers as individually informative. We can do so despite the computational explosion of numbers of possible sets of subsets precisely because CALF uses a greedy approach. Successful permutation tests and other tests then provide model quality assessment.

Diagnosis using new data from a first clinical presentation or understanding where on a continuum of risk a new presentation lies could use historical data that furnishes a weighted sum of chosen predictors. The qualitative metrics CALF uses would be insensitive to multiplication of the computed weight vector by any positive number. Basic CALF finds a direction in p-space, and this is why the coarse coefficients are sufficient.

Regarding again collinearity, suppose several random-valued predictors are added as columns to the data matrix (thus creating perfect coll. Rerunning CALF with the same limit on its number of nonzero weights generally yields a different solution with a mix of the meaningful and meaningless predictors and superior metric value. However, permutation tests will generally yield inferior empirical p-values and loss of a “cliff” in subset popularities, pointing to the importance of using multiple levels of analysis in selection of a classifier. Finding excellent metric performance in itself proves nothing.

## Implementation

### R version

An implementation of the CALF algorithm in the R language is available through the Comprehensive R Archive Network (CRAN) as package CALF implemented as the function calf(). The calf()function may be run with a binary or nonbinary response vector (targetVector). In the binary case, calf() seeks to optimize Welch t-statistic p-value or AUC (optimize = pval or optimize = AUC). Due to the symmetries of those optimizations, the algorithm chooses the initial weight to be + 1. According to user preference of control = 0, case = 1 or the opposite, the weights in the final sum may be reversed. For subsequent MSE optimization, a simple linear transformation may be applied (which of course does not alter p-value or AUC performance).

Alternatively, for a response vector that is real-valued, nonbinary calf() is employed for a positive Pearson correlation using optimize = corr (correlation). Again, a linear transformation may subsequently be applied to minimize MSE (but preserve correlation). The initial weight might be + 1 or − 1. The CALF User Guide fully documents this binary versus nonbinary difference as well as other aspects of the calf() function.

Four supplementary functions are also provided. Permutation tests randomly permute entries in the response vector to reveal the empirical p-value. CALF may be applied to many random subsets (of one or other fixed fraction of all subjects) to find the most "popular" predictors, displaying tables of choices and performance values. Another function cv.calf() enables cross-validation, repeated and/or stratified. For binary response data it selects random subsets of control data of fixed proportion and random subsets of case data of the same proportion; for continuous response data, it selects a fixed proportion of all data. Then cross-validation computes CALF weights and applies the resulting weighted sum to the complementary set. Documentation for these supplementary functions is included in the CALF User Guide in the package. The function perm_target_cv() will conduct the same procedure as cross-validation, documented above, however it will permute the target column (response vector) of the data as a very first step, usually the first column, with each iteration of the process.

Presently, our scope for CALF was simply to provide an accurate implementation of the CALF algorithm plus common methods of evaluation of regression sums. There certainly is room for improvements and enhancements, but changes will include support of the current functional interfaces; thus, there should be no function deprecation with future versions. Further, source code may be minimized such that existing redundant functionality will be moved to a single function, thus making code more concise and maintenance more streamlined. An important future goal is a version that is conformant to popular R statistical modeling packages, especially caret.

### Python version

A Python implementation of CALF obtainable via the PyPi repository at https://pypi.org/project/calfpy/. In a Python environment, installation follows typing pip install calfpy at the command line. The Python version functions in the same manner as the R version, as described above. Given that Python does not natively offer a data frame structure or mechanisms to operate on data frames, the Python CALF implementation relies upon the pandas, numpy, and scipy packages to handle such.

Some hallmark programming techniques often employed in Python are only minimally used in this implementation, e.g., list comprehension. The purpose for this break from style was two-fold: to ensure non-Python programmers could more easily review the code, if desired; and to ensure the code remains somewhat in step with the existing R version. As the core processing is mainly done by the packages listed above, it is not believed these style changes affect performance significantly.

## Supplementary Information

Supplementary Information 1.

Supplementary Information 2.

Supplementary Information 3.

Supplementary Information 4.

## Data Availability

Sample data that may be used for duplication of all the stated results is available via GitHub as an unrestricted supporting resource at https://github.com/jorufo/CALF_SupportingResources. R version: Comprehensive R Archive (CRAN): https://cran.r-project.org/web/packages/CALF/index.html. Python 3.x version: GitHub: https://github.com/jorufo/CALF_Python.
