# Supplementary material for: A greedy regression algorithm with coarse weights offers novel advantages
Source: Sci Rep. 2022 Mar 31;12:5440. doi: 10.1038/s41598-022-09415-2 (PMC8971398; doi:10.1038/s41598-022-09415-2)
Supplement: Supplementary file 2 — Supplementary Information 2. [file 41598_2022_9415_MOESM2_ESM.docx]

Supplement 2: Collinearity

In linear regression, collinearity or near collinearity mean a predictor is equal to or nearly equal to a linear function of other predictors. In collinearity, the moment matrix (transpose of data matrix times itself) cannot be inverted, and the method of ordinary least squares (OLS) fails.

Some other methods also fail or become unstable (e.g., weights might attain large magnitudes and change dramatically with small changes in input values). This well-known difficulty has led to many special modifications. As stated in the article, versions of LASSO logistic regression itself have been specially created in response to collinearity.

This supplement merely illustrates CALF and LASSO logistic regression behaviors with respect to the first example in the article. The other examples yield analogous illustrations but are omitted for the sake of brevity.

As also noted in the article, if certain predictors are linearly dependent or nearly so, CALF simply chooses at most one (the most useful, if any, in terms of improving the metric) and ignores the others. From the data matrix for example 1, we generates two larger data matrices as follows. A “double” data (d) matrix is simply two copies of the true data matrix (so double the number of predictor columns). We designate the second copies of the predictors with a prefix “d”; for example, dMMP7 is the copy of MMP7. In a “double with perturbation” (dp) matrix the predictors in the second copy of the data matrix have all been independently multiplied by a uniformly random number in the range [0.99, 1.01]. We designate the second copies of these predictors with a prefix “dp”; for example, dpMMP7 is the perturbed copy of MMP7. The two new data matrices are shown in Fig. S2.1.


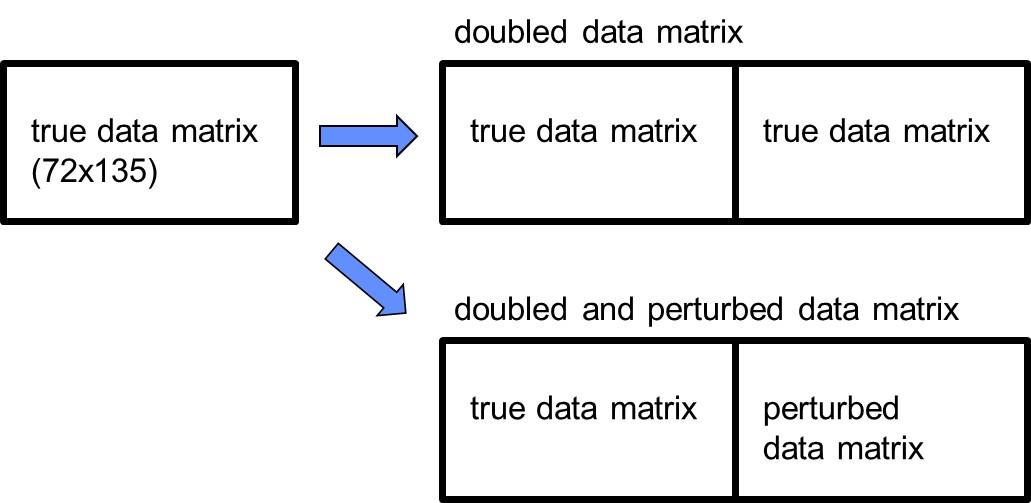


Figure S2.1. The true data matrix of Example 1 was used to generate a data matrix with perfect collinearity of predictors (doubled data matrix) and a nearly collinear matrix of predictors (doubled and perturbed data matrix).

Applying CALF5 with metric = pval to examples of the data matrices we generated yielded the following.

CALF5 applied to true data (as shown in the article)

+MMP7 +MDA-LDL -MMP1 +TSHB -CXCL10

p-value = 2.55E-9

CALF5 applied to doubled data:

+MMP7 +MDA-LDL -MMP1 +TSHB -CXCL10

p-value = 2.55E-9

CALF5 applied to doubled data with small perturbations:

+MMP7 +dpMDA-LDL -dpMMP1 +TSHB -dpCXCL10

p-value = 2.41E-9

We see that CALF5 ignored the doubled predictors in the doubled data matrix. In the dp matrix, however, some perturbed predictors were, by chance, somewhat more useful in improving the metric (pval) and so were selected instead. This resulted in a slightly improved pval for the CALF5 solution.

The application of LASSO logistic regression to the same matrices proceeded as follows. As described in the article, for true data, the value s = 0.075 yielded about the same AUC as CALF5 and so was used.

LASSO.075 applied to true data (as shown in the article; for brevity only a few decimal places of calculated weights are shown):

-0.417 +0.494*MMP7 +0.237*MDA-LDL -0.228*MMP1 +0.184*TSHB -0.181*CXCL10 +0.159*FTL +0.076*CCL8 +0.054*IGHE +0.037*APOD +0.024*KITLG +0.011*TTR -0.0014*IL6 +0.00058*IL1B

MSE = 0.6628

LASSO.075 applied to doubled data matrix:

-0.417 +0. 397*MMP7 +0. 228*MDA-LDL -0.197*MMP1 -0.181*CXCL10 +0. 159*FTL +0. 131*TSHB +0. 097*dMMP7 +0. 071*CCL8 +0. 0536*dTSHB +0. 048*IGHE +0. 037*APOD -0.0304*dMMP1 +0. 024*KITLG +0. 011*TTR +9.09E-3*dMDA-LDL +6.53E-3*dIGHE +5.09E-3*dCCL8 -8.61E-3*IL6 -6.02E-4*dCXCL10 +5.46E-4*IL1B -5.03E-4*dIL6 +3.45E-5*dIL1B +6.32E-6*dTTR +2.65E-6*dKITLG

MSE = 0.6629

Note that many more nonzero weights are employed (24 vs, 13) and that the MSE is very slightly worse.

LASSO.075 applied to doubled data with small perturbations:

-0.417 +0.495*MMP7 +0.239*dpMDA-LDL -0.230*dpMMP1 +0.184*TSHB -0.178*CXCL10 +0.146*FTL +0.078*dpCCL8 +0.055*dpIGHE +0.037*APOD +0.017*KITLG +0.012*dpFTL +0.011*TTR +6.03E-3*dpKITLG -3.18E-3*dpCXCL10 -1.22E-3*dpIL6 +5.68E-4*IL1B +6.31E-5*IL6

MSE = 0.6634

Again, more nonzero weights were employed (17 vs, 13) and the MSE is worse.

The above calculations illustrate that CALF responds predictably and stably to collinearity while LASSO logistic regression does not.
