# Supplementary material for: A greedy regression algorithm with coarse weights offers novel advantages
Source: Sci Rep. 2022 Mar 31;12:5440. doi: 10.1038/s41598-022-09415-2 (PMC8971398; doi:10.1038/s41598-022-09415-2)
Supplement: Supplementary file 3 — Supplementary Information 3. [file 41598_2022_9415_MOESM3_ESM.docx]

Supplement 3. Application of selected conventional models to a binary classification problem

The purpose of this supplement is to compare CALF performance using example 1 vs. performances of several other algorithms. For the other algorithms, default values or a limited set of tuned parameters were used. It is beyond the present scope of work to investigate and attempt to optimize all the parameters of all the other algorithms.

That is, in addition to CALF and LASSO as described in the text, this supplement displays results of several classification schemes that were applied to the same example (135 normalized blood analytes as measured in 40 controls and 32 cases; see <https://github.com/jorufo/CALF_SupportingResources>).

For each of the algorithms applied below, zero, one or two model parameters were optimized using a simple 10000 cross validation (CV) procedure. Specifically, 20% validation hold out sets (8 of 40 controls and 6 of 32 cases) were generated 10000 times. Depending on the model, either a dense grid-search strategy or a random (statistical) search was performed. For each of the chosen models, the estimated parameters and the mean accuracy metric for the CV using the best parameters estimates are reported. Default values were used for all other parameters. The results of using only five most important predictors for the model are also reported since, by comparison, CALF selected five of 135 predictors with optimized empirical p-value. Depending on the model, the predictors may be actual coefficients or estimates of importance.

To test the significance of the fit beyond the 10000 CV experiments, a permutation test was performed using the (crudely) optimized estimated parameters. The permutation procedure was executed 10000 times. The associated empirical p-values^1^ are reported in the following tables. These provide an estimate of the likelihood that the performance of the selected version of the classifier is due merely to chance. Also, the value of the AUC score is reported for each model.

Models: Basic usage

All the following models were accessed through the Python package Scikit-learn^2^.

1) A Logistic Regression Classification model using an elastic net penalty function was tested.^3^

The elastic net penalty parameter L1_ratio was optimized using a discrete grid search in the range (0, 1).

2) A second Logistic Regression Classifier using a pure Lasso penalty^4^ was also tested using default parameters.

3) A Random Forest Classifier (RFN) model^5^ was applied. The only parameter optimized using a grid searching scheme was the number of estimators.

4) A linear Support Vector Machine (SVM) model^2^ was applied. A randomize search was used to estimate the parameters gamma (reciprocal sampling: 0.001 to 0.1) and the scale factor C (uniform sampling 1 to 10).

5) A KNN model^6^ was applied with grid search optimization of the leaf size (5,40) and the number of neighbors (10,70).

6) GaussianNB as in Scikit-learn^2^ using all default parameters was applied.

7) Decision Tree^7^ using all default parameters was applied.

8) Lastly, a hard voting consensus^8^ as available from Scikit-learn was constructed from all the above models.

None of the models using all 135 predictors showed significant fit quality based on the CV scores and the AUC of ROC curves. A simple consensus method (hard voting) of all the tests also failed. A summary appears in Table 1.

Table 1. Selected results for a suite of models applied to the binary classifier problem of our Example 1.

| Model (parameters) | Score (%) | Perm p-value | AUC |
| --- | --- | --- | --- |
| Logistic Regression (elastic net) (alpha=0.0) | 58.57 | 0.13 | 55.70 |
| SVC linear(C=4.75, gamma=0.71) | 54.29 | 0.29 | 58.20 |
| Logistic Regression (purely lasso) | 50.00 | 0.52 | 54.29 |
| Random Forest (estimators=10) | 51.79 | 0.86 | 45.54 |
| KNN (leaf size=10, neighbors=10) | 62.86 | 0.02 | 51.56 |
| Gaussian NB | 41.79 | 0.88 | 40.93 |
| Decision Tree | 51.61 | 0.54 | 45.62 |
| Hard voting consensus | 53.04 | NA | NA |

PCA and ICA predictor set reduction

To examine the importance of reducing the dataset dimension prior to application of the models, both Principle Component Analysis^9^ (PCA) and Independent Component Analysis^10^ (ICA) preprocessing were performed. For ICA, the predictors were whitened. The PCA and ICA methods were accessed from the Scikit-learn package. For both approaches, a total of 5 components were requested. From these, a set of models were applied including the parameter optimization step, followed by permutation testing and the AUC-ROC determination. The results are collected in the following Tables. Generally, reduction of the components had little effect on the results. In all cases poor classifier performance continued to be observed. In the following plot are the PCA eigenvalues. Clearly, reduction of predictors based on either variance (PCA) or information (ICA, up to 500 iterations) is insufficient to build a quality model for this small data set.

Table 2. Principal component analysis (PCA) with five components specified.

| Model (parameters) | Score (%) | Perm p-value | AUC |
| --- | --- | --- | --- |
| Logistic Regression (elastic net, alpha=0.9) | 57.14 | 0.251 | 0.593 |
| Logistic Regression (LASSO) | 55.89 | 0.315 | 0.489 |
| Random Forest (estimators = 24) | 50.00 | 0.739 | 0.496 |
| Decision Tree | 41.61 | 0.933 | 0.402 |
| Hard voting consensus | 57.32 | NA | 0.403 |

The PCA eigenvalue spectrum indicates that the amount of variance explained when including only 5 components is rather small, ranging from 0.105 down to 0.048.

In the next Table 3 are analogous tests using Independent Component Analysis (ICA) for predictor set reduction.

Table 3. Independent component analysis (ICA) using five components

| Model (parameters) | Score (%) | Perm p-value | AUC |
| --- | --- | --- | --- |
| Logistic Regression (elastic net, alpha=0.1) | 55.71 | 0.251 | 0.372 |
| Logistic Regression (LASSO) | 55.71 | 0.315 | 0.491 |
| Random Forest (estimators = 24) | 50.00 | 0.739 | 0.430 |
| Decision Tree | 38.57 | 0.933 | 0.534 |
| Hard voting consensus | 52.86 | NA | NA |

Limited predictor set computation

A (biased) test of the five best predictors as selected by the CALF solution on 100% of true data (CALF5 = +MMP7 +MDA-LDL - MMP1 +TSHB - CXCL10) was also performed using several of the tested traditional models. In all cases substantially better CV scores, permutation p-values, and AUC-ROC were observed. This suggests that the predictors identified by CALF do indeed capture relevant association of predictors and the target.

Table 4. Using the five (best) CALF predictors only

| Model (parameters) | Score (%) | Perm p-value | AUC |
| --- | --- | --- | --- |
| Logistic Regression (elastic net, alpha=0.0) | 76.43 | 0.0002 | 0.834 |
| Logistic Regression (LASSO) | 76.43 | 0.0001 | 0.827 |
| Random Forest (estimators = 19) | 66.43 | 0.0009 | 0.695 |
| Decision Tree | 57.14 | 0.364 | 0.603 |
| Hard voting consensus | 76.43 | NA | NA |

By comparison, the AUC of CALF5 exceeds all the above values. The ROC is shown in Figure 1.

Figure 1. ROC of a Logistic Regression model built with the five predictors selected by CALF.

References

1 North, B. V., Curtis, D. & Sham, P. C. A note on calculation of empirical P values from Monte Carlo procedure. *Am J Hum Genet* **72**, 498-499 (2003).

2 Pedregosa, A. F. *et al.* Scikit-learn: Machine Learning in Python. *Journal of Machine Learning Research* **12**, 2825-2830 (2011).

3 Zou, H. & Hastie, T. Regularization and variable selection via the elastic net. *J R Stat Soc B* **67**, 301-320, doi:DOI 10.1111/j.1467-9868.2005.00503.x (2005).

4 Tibshirani, R. The lasso method for variable selection in the Cox model. *Stat Med* **16**, 385-395 (1997).

5 Breiman, L. Random Forests. *Machine Learning* **45**, 5-32 (2001).

6 Bentley, J. L. Multidimensional Binary Search Trees Used for Associative Searching. *Commun Acm* **18**, 509-517, doi:Doi 10.1145/361002.361007 (1975).

7 Hastie, T., Tibshirani, R. & Friedman, J. *The Elements of Stastistical Learning: Data Mining, Inference, and Prediction, Second Edition*. (Springer, 2009).

8 Wolpert, D. H. Stacked Generalization. *Neural Networks* **5**, 241-259, doi:Doi 10.1016/S0893-6080(05)80023-1 (1992).

9 Tipping, M. E. & Bishop, C. M. Probabilistic principal component analysis. *J R Stat Soc B* **61**, 611-622, doi:Doi 10.1111/1467-9868.00196 (1999).

10 Hyvarinen, A. & Oja, E. Independent component analysis: algorithms and applications. *Neural Networks* **13**, 411-430, doi:Doi 10.1016/S0893-6080(00)00026-5 (2000).
