# Supplementary material for: A greedy regression algorithm with coarse weights offers novel advantages
Source: Sci Rep. 2022 Mar 31;12:5440. doi: 10.1038/s41598-022-09415-2 (PMC8971398; doi:10.1038/s41598-022-09415-2)
Supplement: Supplementary file 4 — Supplementary Information 4. [file 41598_2022_9415_MOESM4_ESM.docx]

Supplement 4. Computational complexity

The purpose of this supplement is estimate CALF computational cost using data matrices constructed from example 1. As in Supplement 2, the true data matrix (72X135) with small perturbations was used to make a synthetic data matrix with twice as many predictors (72X270). This process was continued to synthesize 72X405, 72X540, and 72X675 matrices. This construction is shown in Fig. S4.1.


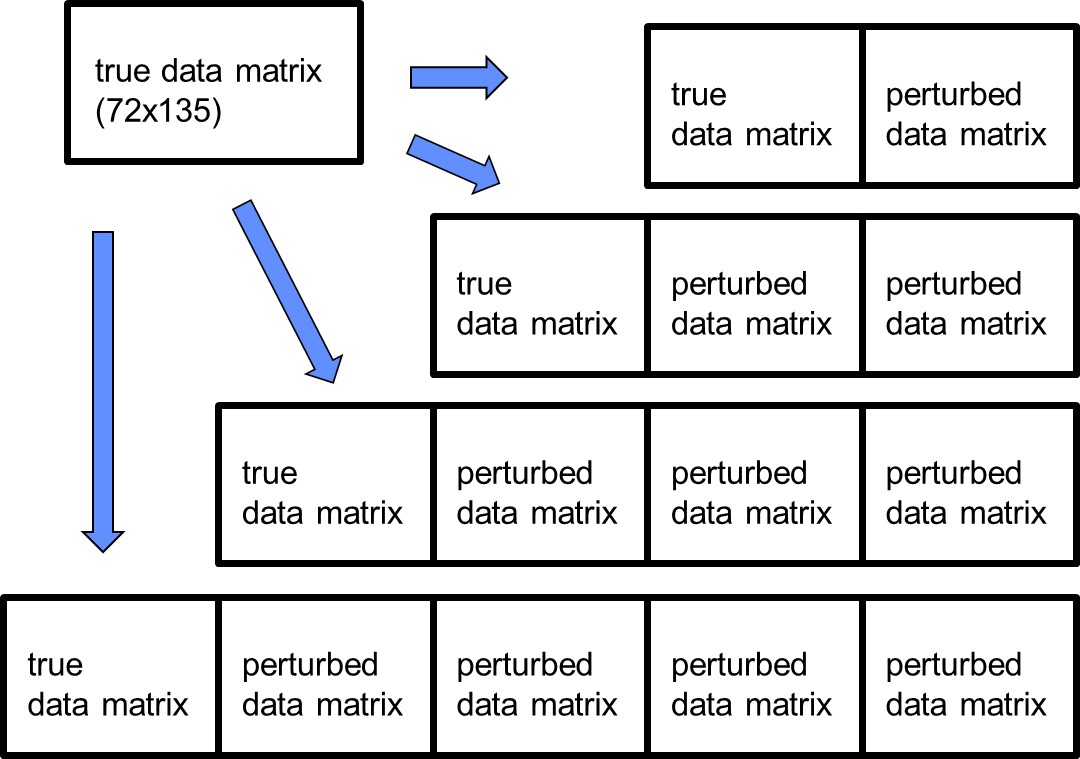


Figure S4.1. A true data matrix with multiple generations of perturbations was used to synthesize larger data matrices for the purpose of evaluation computational cost.

For p predictors and a limit of L selection, the CALF algorithm must execute up to (2*p+1-L)*L/2 loops through them.

We programmed CALF for 100 permutation test calculations using L = 20 and the pval metric. We timed completion of the permutations on a laptop. The results are shown in Fig. S4.2.


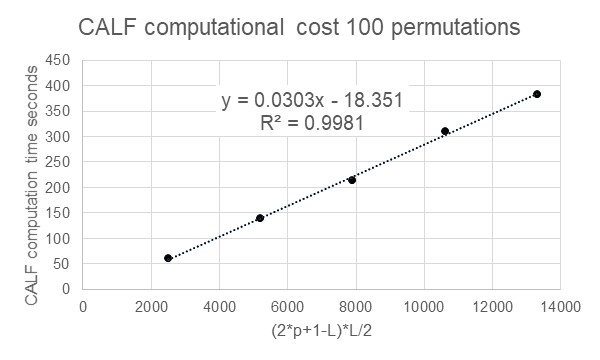


Figure S4.2. CALF was applied to the five matrices in Fig. S4.1 and looped 100X through permutation tests with L = 20. The time costs were recorded. Shown is a plot of the time costs as a function of the number of loops.

Lastly, we generated a 72X9312 matrix (response vector as in Example 1, number predictors as in Example 5) to extend the above results. CALF20 was applied to these synthetic data, and 7380 seconds (about 2.05 hours) were required for 100 permutation tests. Adding this datum to the above study produces Fig. S4.3.


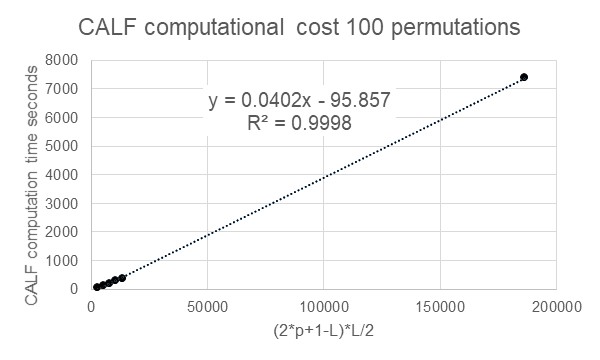


Figure S4.3. Adding a datum for one much larger data matrix yields approximation by a similar linear time cost.

We conclude that for L<<p, the computational cost of CALF is of order O(p*L).

Of course, lengthy permutation or subset jobs should be parallelized. The effect of using x nodes would be nearly division of time cost by x.
